# Supplementary material for: Extractability of Curcuminoids Is Enhanced with Milk and Aqueous-Alcohol Mixtures
Source: Molecules. 2022 Jul 30;27(15):4883. doi: 10.3390/molecules27154883 (PMC9369953; doi:10.3390/molecules27154883)
Supplement: Supplementary file 1 [file molecules-27-04883-s001.zip › molecules-1778753-supplementary.pdf]

*Supplementary Materials*

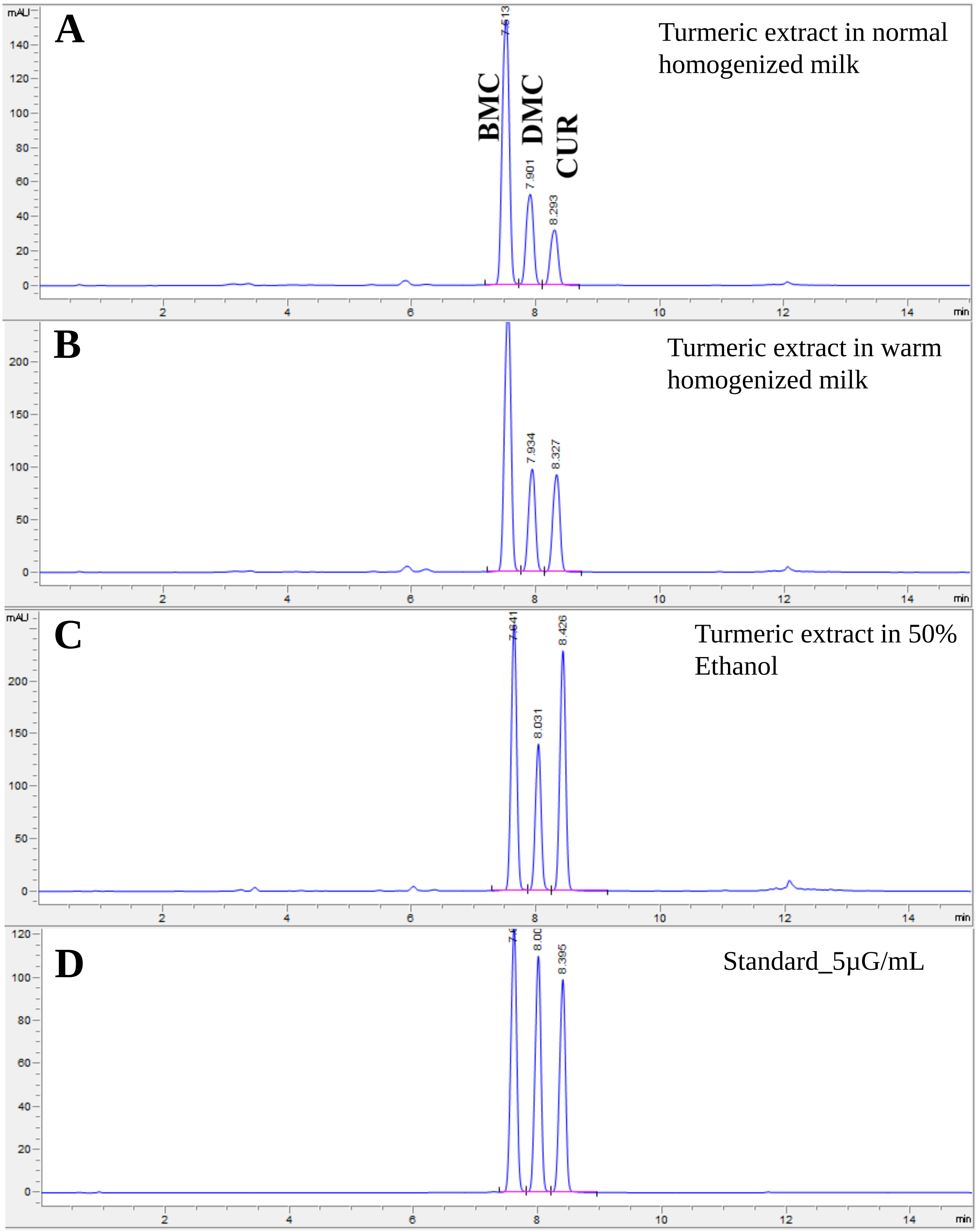


**Figure S1.** Representative HPLC-DAD chromatogram of curcuminoids CUR, DMC, and BMC from Turmeric extract in A. in normal homogenized milk, B. warm homogenized milk, and 50% aqueous ethanol compared to D. the standard mixture (5 μg/mL).

**Phenolic Content Analysis.**

The total phenolic content was analyzed using the FC assay, as described previously.[1,2] In brief, 20 μL of turmeric extracts was pipetted into a 96-well plate and diluted with 177 μL of deionized water. This mixture was shaken gently for 30 sec. The FC reagent

(12.5 μL) was added to each well and incubated for 8 min in the dark at room temperature. Next, 20% sodium carbonate (37.5 μL) was added to each well and mixed gently. The mixture was incubated for another 2 h in the dark. After 2 h, the reaction products' absorbance was measured using a Spectramax 384 Plus microplate reader from Molecular Devices (Sunnyvale, CA) at 765 nm. Gallic acid with concentrations of 0, 0.25, 0.5, 0.75, 1.0, and 1.75 mg/mL was used as the standard for the calibration curve. The phenolic content was calculated based on the calibration curve with linearity $r^2 = 0.9967$. Results were expressed in milligrams of gallic acid equivalent per gram (mg of GAE/g) of turmeric.

**Study of Antioxidant Activity.**

The antioxidant capacity of the turmeric extract was performed with a previously reported FRAP assay, with some modifications.[2,3] A mixture of 2,4,6-tris(2 pyridyl)-s-triazine (TPTZ) (10 mM in 40 mM HCl), sodium acetate buffer (300 mM, pH 3.6), and ferric chloride hexahydrate (20 mM) with a ratio 1:10:1(v/v/v) was used as FRAP working solution. The 6-hydroxy-2,5,7,8-tetramethyl-chroman-2-carboxylic acid (TROLOX) was used as a standard for the calibration curve. The calibration curve was obtained from five concentrations (0, 0.05, 0.1, 0.2, 0.3, and 0.5 mM) of the TROLOX standard solutions. Next, 20 μL of turmeric extract (20 mg/mL) was taken into a 96-well microplate. To each well, 230 $\mu$L FRAP working solutions were added and mixed gently. The reaction mixture was placed in the dark and incubated for 10 min at room temperature. The absorption of the reaction product was measured at 595 nm using a Spectramax 384 Plus microplate reader from Molecular Devices. Results were expressed in milligrams of Trolox equivalent per gram (mg of TE/g) of turmeric.

**Correlation Studies Between Colorimetric Assays (Folin–Ciocalteu (FC) and Ferric Reducing Antioxidant Power (FRAP)) and HPLC Analysis.**

Figures S1A and S1B show a correlation between HPLC analysis of curcuminoids with diode-array detection and the two colorimetric assays in different aqueous-alcohol solutions. The results show a good correlation between the two sets of samples with $r^2$ values of 0.98 (HPLC vs. FC) and 0.95 (HPLC vs. FRAP). However, the correlation was significantly reduced ($r^2$ 0.70 for HPLC vs. FC and 0.54 for HPLC vs. FRAP) when milk samples were used. This is due to the presence of other components, including lipids and other biomolecules, in the milk matrices. This study emphasizes that traditional assays such as FC and FRAP antioxidant assays may not be suitable for milk samples due to the inherent experimental limitations associated with the matrix effects. This further justifies that the conclusions drawn from different colorimetric assays used to measure total phenolic content and various bioactivities, such as antioxidant activity, should always be confirmed with secondary chromatography methods to avoid variations in values reported by a different researcher in foods or dietary supplements.

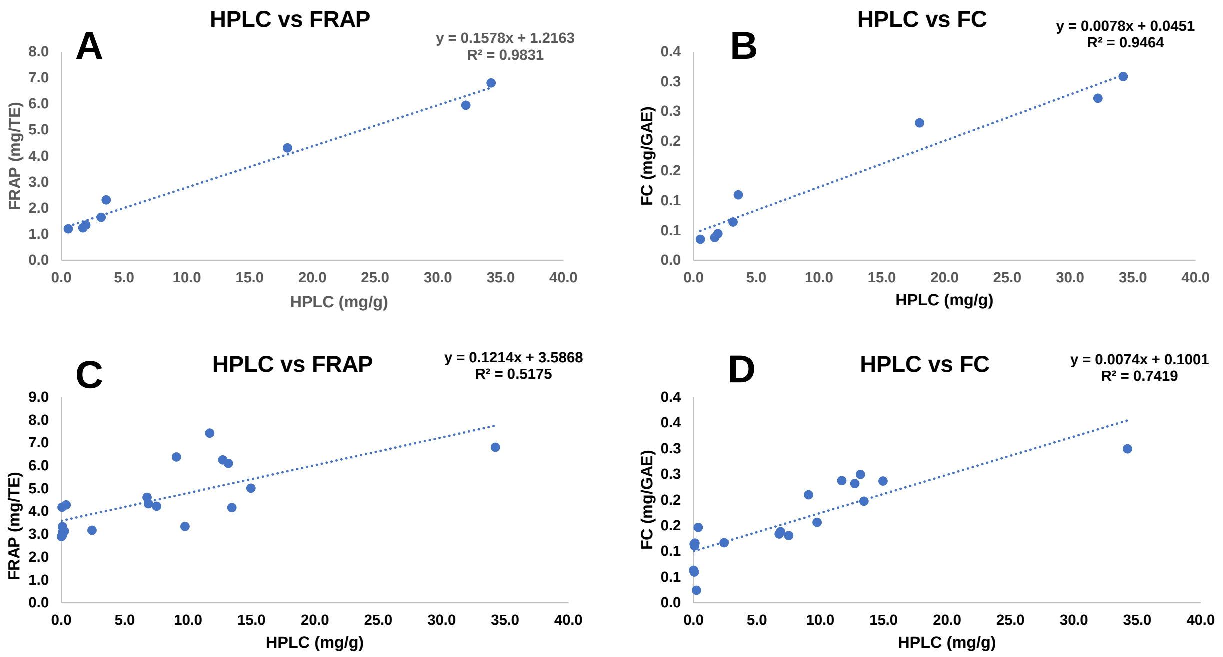


**Figure S2.** Correlation studies of HPLC data with antioxidant assay results; curcuminoids extracted in aq. alcohol solutions **A**. HPLC vs. FRAP, **B**. HPLC vs. FC; and curcuminoids extracted in different milks; **C**. HPLC vs. FRAP, and **D**. HPLC vs. FC.

## References


29. Luthria, D.L.; Mukhopadhyay, S.; Krizek, D.T. Content of Total Phenolics and Phenolic Acids in Tomato (Lycopersicon Esculentum Mill.) Fruits as Influenced by Cultivar and Solar UV Radiation. *J. Food Compos. Anal.* **2006**, *19*, 771–777. https://doi.org/10.1016/j.jfca.2006.04.005.
30. Tareq, F.S.; Kotha, R.R.; Ferreira, J.F.S.; Sandhu, D.; Luthria, D.L. Influence of Moderate to High Salinity on the Phytochemical Profiles of Two Salinity-Tolerant Spinach Genotypes. *ACS Food Sci. Technol.* **2021**, *1*, 205–214. https://doi.org/10.1021/acsfoodscitech.0c00034.
31. Luthria, D.L. A Simplified UV Spectral Scan Method for the Estimation of Phenolic Acids and Antioxidant Capacity in Eggplant Pulp Extracts. *J. Funct. Foods* **2012**, *4*, 238–242. https://doi.org/10.1016/j.jff.2011.11.002.
